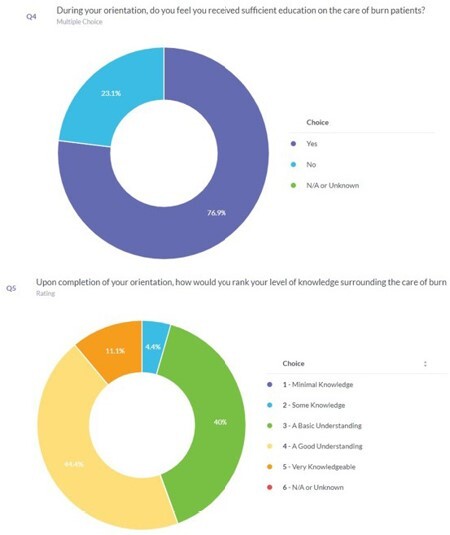# 555 Implementing BNCI Guided Burn Nurse Competency Education at Kessler Burn Trauma Step down Unit

**DOI:** 10.1093/jbcr/irae036.189

**Published:** 2024-04-17

**Authors:** Rhoda M Palacio, Holly Moynihan

**Affiliations:** University of Rochester Medical Center, Strong Memorial Hospital, Rochester, NY; University of Rochester Medical Center, Strong Memorial Hospital, Rochester, NY

## Abstract

**Introduction:**

The Burn Nurse Competency Initiative (BNCI) at the American Burn Association developed a robust Burn Nurse competency national standard that shaped the culture and competence of burn nurses across the country. Upon close analysis of our current practice, it was determined that there are no competency guidelines specific to burn nursing care available for nursing staff during the on-boarding process as well as annual nursing competency assessment available to maintain and sustain staff’s learning needs. The development and implementation EBP Burn Nurse Competencies (BNC) education and its application into our current practice is an important quality improvement initiative that will greatly improve the current standard of care, provide standardization in the process of orientation, and enable sustainable learning opportunities to all nurses in a verified burn center, as well as improve treatments and therapies from admission to discharge that will allow the best possible outcomes for our burn patients.

**Methods:**

The initial needs assessment survey was sent out to our current staff prior implementation of BNC education. The result has shown 76% of staff feels that they received sufficient education on the care of burn patients. The BNC education was also developed through utilization of the cumulative input from the burn program interdisciplinary team and integration of their individual department’s standard of care for burn patients. The BNC was created with two parts: a comprehensive burn/wound care management and the BNC education which defines the specific domain of BNCI competency standard. All of the eleven domains were also integrated in the performance criteria assessment tool. This tool was developed and utilized to ascertain knowledge gained and also as a means to validate competence and confidence level of nurses as they apply the skills/knowledge gain in our clinical setting. The BNC education was implemented to the current staff and is also currently being used to onboard newly hired nurses as the foundation of their orientation process.

**Results:**

The results of this evidence-based practice quality improvement project will be gathered and analyzed at the end of this year when all current newly hired RN will be off orientation.

**Conclusions:**

The conclusion of this quality improvement project will be analyzed at the end of the year for our new hired RN’s orientation process. A survey will be sent out to these group of nurses to ascertain the effectiveness of the BNC education which was implemented July of this year.

**Applicability of Research to Practice:**

Research studies have shown the significance of identifying practice gap related to nursing competencies in practice, assessing the need of nursing competencies, the development of accompanying educational tool and evaluating the effectiveness of nursing competencies as crucial in preventing poor patient outcome, near-miss events, and preventable hospital-acquired conditions.